# Ossicular Erosion in Patients Requiring Surgery for Cholesteatoma

**Published:** 2012

**Authors:** Ghodrat Mohammadi, Masoud Naderpour, Mehrnoosh Mousaviagdas

**Affiliations:** 1 *Department of otorhinolaryngology, Tabriz University of Medical Sciences, Tabriz, Iran*

**Keywords:** Cholesteatoma, Chronic, Erosion, Ossicular, Otitis media

## Abstract

**Introduction:**

The aim of this study was to evaluate the condition of the ossicular chain in patients requiring surgery for cholesteatoma.

**Materials and Methods:**

In a retrospective analysis, the destruction of the individual and combined bony structures of the ear was described in 166 patients with cholesteatoma who went through surgery in our Otology Center between 2003 and 2009.

**Results:**

Total (55.4%) or partial (30.7%) erosion of the incus was the most common pathology. In some cases, the long process (25.9%) and the body of incus (4.8%) were also involved. Erosion of the stapes superstructure occurred more commonly than a total loss of the bone (40.9% vs. 25.9%). Erosion of the malleus was least common. Completely intact ossicles were present in 5.5% of cases. Total ossicular erosion with an intact footplate (18.7%) and incudostapedial erosion (18%) was the most common combination of ossicular erosion. All patients with incudostapedial erosion had advanced disease (85% with multiple site involvement).

**Conclusion:**

Widespread cholesteatoma results in greater ossicular erosion and poor hearing outcomes.

## Introduction

Otitis media is the most common bacterial disease in children and happens in 80% of children between the ages of one and six years, and it is the disease most frequently managed with antibiotics. But later in life, in some of these patients may result in serious morbidity and complication, such as, Cholesteatoma and ossicular erosion (1).

Cholesteatoma is an expanding destructive disease of the temporal bone that is composed of stratified squamous epithelium. This disease is often developed in the mastoid and middle ear but it can form in every part of the temporal bone (2). Despite a decrease in the incidence of chronic otitis media, it is still a major cause of considerable morbidity (3).

Chronic otitis media can cause disruption in the continuity of the ossicular chain, with the most damage being caused when cholesteatoma is also present (4). This pathology may be limited to the incudostapedial joint, but usually total or partial loss of the long process of the incus can be seen as well. An important factor in the decision to use prosthetics for bone restoration in these cases is the state of the bones.

The present study was conducted to evaluate the state of the ossicular bones in patients who require surgery for cholesteatoma, so that the findings can be used to predict the need for bone reconstruction and determine the choice of prosthesis preoperatively. Furthermore, the results will also increase general health care knowledge and prevent delayed referral of patients with chronic otitis media.

## Materials and Methods

 A total of 166 patients suffering from chronic otitis media with cholesteatoma that was resistant to treatment with systemic and local antibiotics were operated on during a 6-year period (2003–2009). Patients with diabetes, immune deficiency, vasculopathy, or who had undergone radiotherapy for any reason were excluded from the study. All patients underwent a canal wall down mastoidectomy. During the operation all the ossicles were analyzed in terms of health, erosion, and total loss. The results were analyzed in a retrospective manner. The details of incomplete erosions are also discussed. The statistical significance of differences between paired groups (such as sex) was tested using the chi-squared test and significance was defined as P< 0.05. 

## Results

A total of 166 patients with a mean age of 30.8 years (range: 5–69 years), all of whom had chronic otitis media with cholesteatoma were the subjects of this study. The majority of the patients were within an age range of 21 to 30 years old, and overall most patients were younger than 30 (Table 1); 48.2% of the patients were female and 51.8% were male. 

**Table 1:** Age of patients with chronic otitis media

**Age**	**Number of cases (%)**
**5–10 years** **11–20 years** **21–30 years** **31–40 years** **Greater than 40 years** **Total**	**3 (1.8)** **41 (24.7)** **48 (28.6)** **36 (21.6)** **38 (23)** **166 (100)**

It seems that the gender of the patients had no effect on the occurrence of their condition (P>0.05). The results of the evaluation of ossicular damage are reported in (Table 2).

Out of the 166 patients, 157 (94.5%) were affected by erosion of the ossicles. Involvement of the incus was most common (86.1%), followed by involvement of the stapes (66.8%), and then the malleus (43.9%).Overall, the most common pattern of damage was observed in the incus bone,which included total loss in 55.4% of cases and partial damage in 30.7%.

**Table 2:** State of the middle ear bones in patients with chronic otitis media with cholesteatoma as determined during surgery

**Ossicle**	**Surgical findings**	**Number of cases (%)**
**Malleus**	Intact Eroded Total loss	93 (56.0) 37 (22.3) 36 (21.7)
**Incus**	Intact Eroded Total loss	23 (13.8) 51 (30.7) 92 (55.4)
**Stapes superstructure**	Intact Eroded Total loss	98 (59.0) 68 (40.9) 43 (25.9)

The most widely damaged part of the incus was its long process (25.9%) and the second most common site of damage to the incus was its body (4.8%). Damage to the stapes was the second most common type of damage observed, and mostly involved its superstructure. Erosion of the stapes superstructure was more common than total loss of the bone (40.9% vs. 25.9%, respectively). Even though damage to the malleus was least common, the number of cases where the stapes superstructure was intact was greater than the number of cases of an intact malleus (59% vs. 56%). 

In only nine patients were all the ossicles intact. The most common combination of erosion of the ossicles was damage to all the ossicles except for the footplate (18.7%). Concurrent damage to the incus and the superstructure of the stapes was the next most common combination of damage (18%). In these cases, complete destruction of the incus with partial damage of the stapes superstructure occurred in equal proportion to partial damage of both the incus and the stapes superstructure (9% each). Partial damage of the malleus and the superstructure of the stapes with complete destruction of the incus was the third most common combination of damage (15.7%). Involvement of the stapes was very common in patients aged below 30 (90% vs. 54%). As just described, the incus was the most commonly damaged ossicle and where damage was observed in other ossicles, the incus was always involved to

some extent. 

Patients suffering from damage to all three ossicles also had extensive cholesteatoma in the tympanic, mastoid, attic, and antrum cavities. Most patients who had involvement of multiple ossicles, had significant cholesteatoma inside the tympanic cavity such that 85% of patients with damage to the incus and stapes showed cholesteatoma of the tympanic sinus, oval window, facial recess, and round window.

## Discussion

In our study, 94.5% of patients were suffering from ossicular damage. The majority of these patients had extension of cholesteatoma into the tympanic and mastoid cavities. It seems that the presence of cholesteatoma is a chronic phenomenon and that patients are only referred for treatment in the later stages of the disease, which suggests a lack of information, education, training, and shortage of health care. Our patients were not examined in terms of their hearing abilities, but in a study by Eero and Juhani in 1993 it was concluded that auditory function in ears that had a healthy stapedial bone was better than in cases where there was damage in all three ossicles (5).

The percentage of cases with involvement of each of the osiccles was as follows: incus 86.1%, stapes 66.9%, and malleus 43.9%. In a study by Kurien and colleagues these figures were: incus 100% and malleus 67%, with stapes involvement occurring more in children than in adults (95% vs. 67%) (6). There are some differences between these figures and in our study involvement of the stapes was more prevalent in patients aged below 30 (90% vs. 57%). In another study by Garap and Dubey, involvement of the ossicles was reported as follows: incus 89%, stapes 41%, and malleus 32 % (7). However, a study by Brakeman had different results again and showed that in children all the ossicles were intact in 9% of cases, the malleus and stapes were intact in 11% of cases, and only the stapes was intact in 46% of cases.

In adults these percentages were 5%, 3%, and 73%, respectively (8). The findings regarding the ossicular chain in a study by Palva and colleagues (9) describe a picture that includes more involvement of the ossicular chain in adults than in children. However, our patients were almost all adults but in 18.7% of the patients all the ossicles were damaged. Another study also mentioned that most patients (57.2%) had the widest pattern of cholesteatoma expansion (10). Thus it seems that most patients that were referred to our clinic were in the advanced stages of the disease. According to the high rate of cholesteatoma extension into the mastoid and tympanic cavity in patients with erosion of multiple ossicles, it seems that these patients were experiencing severe cholesteatoma. Once in this severe state they will need wider and more aggressive surgeries to stop the remaining cholesteatoma. 

Thus, due to the extensive damage to the ossicles, the difficulty of restoring the bone during a single procedure, and the probable need for several surgeries, the outcome of attempts to restore hearing can be limited in these patients. Patients must therefore be fully informed about the potential issues before surgery.

## Conclusion

This study shows that despite a decrease in the number of cases of chronic otitis media, there are still cases of extensive cholesteatoma that can cause greater ossicular erosion and poor hearing outcomes. To decrease this ossicular damage, physicians need to be alert and informed about chronic otitis media and the extensive cholesteatoma that causes erosion of the ossicles.
